# Ginsenoside Rb1 Produces Antidepressant-Like Effects in a Chronic Social Defeat Stress Model of Depression Through the BDNF–Trkb Signaling Pathway

**DOI:** 10.3389/fphar.2021.680903

**Published:** 2021-09-29

**Authors:** Ning Jiang, Hong Huang, Yiwen Zhang, Jingwei Lv, Qiong Wang, Qinghu He, Xinmin Liu

**Affiliations:** ^1^ Research Center for Pharmacology and Toxicology, Institute of Medicinal Plant Development (IMPLAD), Chinese Academy of Medical Sciences and Peking Union Medical College, Beijing, China; ^2^ Sino-Pakistan Center on Traditional Chinese Medicine, Hunan University of Medicine, Huaihua, China; ^3^ Affiliated TCM Hospital/School of Pharmacy/Sino-Portugal TCM International Cooperation Center, Southwest Medical University, Luzhou, China

**Keywords:** ginsenoside Rb1, depression, CSDS, BDNF, neurogenesis, mice

## Abstract

Ginsenoside Rb1 (Rb1), an important bioactive ingredient of *Panax ginseng*, has potent neuroprotective effects. The objective of the study is to elucidate the impact of Rb1 treatment on chronic social defeat stress (CSDS)–induced depressive-like behaviors and its related mechanism. According to the obtained results, the daily oral administration of Rb1 (35 and 70 mg/kg) and imipramine (15 mg/kg) for 28 days significantly reversed the social avoidance behavior, anhedonia, and behavioral despair *via* CSDS exposure, as demonstrated by the considerable elevation in the time in the zone in the social interaction test, consumption of sucrose solution in the sucrose preference test, and decrease in immobility time in the forced swim test. Moreover, Rb1 obviously restored the CSDS-induced decrease in the BDNF signaling pathway and hippocampal neurogenesis. Rb1 significantly increased the hippocampal levels of ERK, AKT, and CREB phosphorylation and increased the number of DCX+ cells in DG. Importantly, the antidepressant effects of Rb1 were completely blocked in mice by using K252a (the nonselective tyrosine kinase B inhibitor). In conclusion, our results indicated that Rb1 exerts promising antidepressant-like effects in mice with CSDS-induced depression, and its effects were facilitated by enhancing the BDNF signaling cascade and upregulation of hippocampal neurogenesis.

## Introduction

Stress is substantially involved in many neuropsychiatric complications, such as anxiety, depression, and posttraumatic stress disorders. Depression is a prevalent neuropsychiatric disorder, and its main clinical characteristics are constant and obvious depression of mood. Worldwide, the lifetime prevalence of depression is approximately 15–20%, which means that almost one in five people experience depression in their lifetime, and 15% of cases of major depressive disorder result in suicidal deaths (Malhi and Mann, 2018). During the last few years, the monoamine hypothesis has been one of the most widely studied etiologies of depression. Nearly all currently used antidepressants work by increasing the levels of monoamine neurotransmitters. However, these antidepressant agents usually take weeks to months to exert their therapeutic effects. Furthermore, more than 30% of patients do not respond to these agents (Licinio and Wong, 2005; Fukumoto et al., 2019). Thus, there is a need to develop more effective and safer antidepressants.

To date, various studies have attempted to explore the pathogenesis of depression and develop antidepressant drugs by using animal models subjected to chronic stress (Ito et al., 2017). Chronic social defeat stress (CSDS) is associated with depression in humans and simulates human emotions, such as fear caused by failure and frustration (Von Frijtag et al., 2000). Indeed, environmental stressors associated with daily life (such as a high-pressure social environment) are more common causes of depression than primary neural circuit impairment (Prudo et al., 1981). In the CSDS paradigm, model animals are subjected to psychological social stress *via* a widely adopted preclinical stress procedure with excellent face, constructive, and predictive validity (Hao et al., 2019; Warren et al., 2020). Long-term CSDS causes changes corresponding to the symptoms of post-stress depression, including elevated social avoidance, behavioral despair, and anhedonia, while these CSDS-stimulated behavioral changes can be ameliorated by long-term (10 days) treatment with traditional antidepressant drugs (Robison et al., 2014; Munshi et al., 2020).

According to recent studies, the BDNF signaling cascade is crucial for depression treatment and pathophysiology (Schmidt and Duman, 2007). Through tyrosine kinase B (TrkB), BDNF signaling induces the phosphorylation and activation of CREB. Furthermore, BDNF and TrkB have also been found to significantly activate signaling cascades associated with downstream signaling of BDNF, that is, the AKT and MAPK-ERK signaling cascades (Jiang et al., 2015; Ghosal et al., 2018). It has been extensively shown that continuous exposure to stress decreases the level of hippocampal BDNF in animal models of depression, and BDNF expression has been found to be decreased in the brains of patients with major depressive disorders (Wang et al., 2016; Jiang et al., 2019a). In animal models and clinical studies, several drugs have been found to exert antidepressant-like effects and regulate the expression of proteins associated with BDNF signaling pathways (such as CREB, AKT, and ERK) (Luo et al., 2015).

Traditional herbal medicines have been used for many years and have been proven effective for the treatment of various neuronal disorders, such as depression (Liu et al., 2016). Recently, the use of medicinal plant–based medicines to treat depression has gained wider scientific attention because these drugs have few side effects (Sun et al., 2014). Ginsenoside Rb1 (Rb1) is the most significant bioactive constituent of the herb *Panax ginseng* C.A. Meyer. In Far East countries, this herb has been widely used as a tonic for more than 2000 years. During the last few decades, *Panax ginseng* C.A. Meyer has also gained scientific recognition as a tonic remedy in Western countries (Helms, 2004). Previous pharmacological studies have revealed that Rb1 exerts multiple biological effects, including antioxidant, antitumor, and anti-inflammatory effects. (Deng et al., 2017; Xin et al., 2019). Rb1 has been demonstrated its protection for the central nervous system and is apparently highly distributed to the brain (Wang GL. et al., 2018).

Previous reports have shown that Rb1 exerts a significant antidepressant-like effect in a chronic unpredicted mild stress model by mediating central neurotransmitters of the noradrenergic, serotonergic, dopaminergic, and aminoacidergic systems (Wang et al., 2017; Wang YZ. et al., 2018). Recent studies have revealed that Rb1 alleviates depressive-like behaviors, suppresses neuroinflammation, and activates the AKT pathway in mice subjected to chronic restraint stress (Guo et al., 2021). However, several studies have demonstrated that Rb1 has therapeutic potential against depression-like conditions and that it exerts an antidepressant-like effect against behavioral abnormalities caused by CSDS; however, its underlying mechanism is not fully understood. In the current study, the antidepressant potential of Rb1 was evaluated in CSDS-exposed mice. To further explore the possible mechanism, the role of the BDNF signaling cascade in the hippocampus was also assessed.

## Materials and Methods

### Animals

The CD1 (12 months old, male) and C57BL/6J (7–8 weeks old, male) mice were provided by the Institute of the Chinese Academy of Medical Science Center, Beijing. Both types of mice were kept in the maintained place according to the standard animal housing conditions, such as 55 percent humidity, 20–22°C temperature, and 12:12 light and dark duration, with free access to water and food. The experimental procedure involving animals was performed with proper approval (approval no. SYXK 2017-0020), following the guidelines provided by the Animal Research Committee of the Institute of Medicinal Plant Development, Peking Union Medical College.

### Drugs and Treatments

Ginsenoside Rb1 (HPLC grade, greater than 98% purity) was obtained from Ruifensi Biological Technology Co., Ltd. (Chengdu, China). Imipramine (IMI) was provided by Sigma–Aldrich Co. (St. Louis, MO, United States). All these compounds were administered at a dose of 10 ml per kg of body weight. This study was divided into two experiments.

Experiment 1: Animals were randomly allotted to one of five groups: the control, CSDS-exposed, Rb1-treated (35 or 70 mg per kg of body weight), and IMI-treated groups (15 mg per kg of body weight).
Experiment 2: Animals were randomly allotted to one of five groups: the control, CSDS-exposed, Rb1-treated (70 mg per kg of body weight), IMI-treated (15 mg per kg of body weight), and CSDS + Rb1 (70 mg per kg) +K252a (25 μg per kg)-treated groups.

**FIGURE 1:**
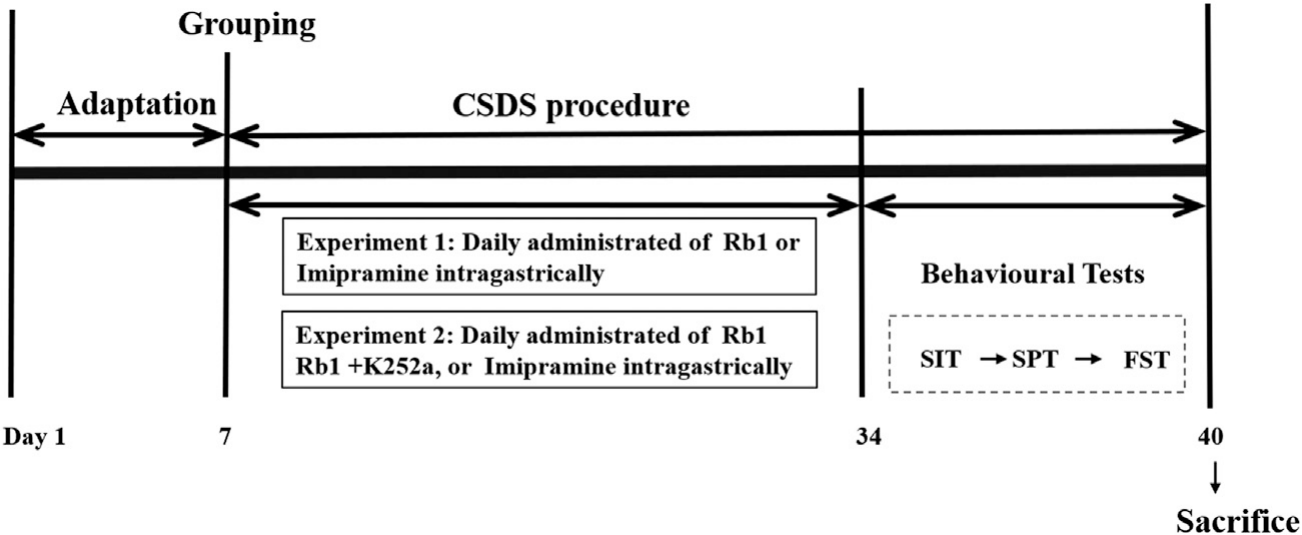
Experimental design. Ginsenoside Rb1, Rb1; chronic social defeat stress, CSDS; social interaction test, SIT; sucrose preference test, SPT; forced swim test, FST.

All mice, except those in the control group, were exposed to CSDS for 38 days. Water, Rb1, or IMI was administered orally to CSDS-exposed mice for 33 consecutive days until the behavioral tests were completed. The experimental procedure is shown in Figure 1.

### CSDS Procedure

CSDS was performed as previously described with minor modifications (Golden et al., 2011; Monleon et al., 2015; Jiang et al., 2020b). In brief, CD1 mice were used to study the aggressive behavior of the CSDS-exposed mice and to evaluate the tendency of the mice to attack a mouse following intrusion into their home cage (Sial et al., 2016). C57BL/6 mice were subjected to physical defeat by exposing them to aggressor CD1 mice for 5 min daily for 28 days. The C57BL/6 mice were housed in the same cage as an aggressor mouse separated *via* a transparent organic acrylic plate (4 mm thick and porous) the following day and were exposed to chronic psychological stress, such as threatening auditory, olfactory, and visual stimuli, for the subsequent 24 h. The control mice were housed in pairs in the same cage separated by an identical porous transparent organic acrylic plate.

### Social Interaction Test

The SIT was carried out according to previously reported methods with slight modifications (Bo et al., 2015). In brief, each mouse was kept in an open-field arena (40 × 40 × 40 cm) containing a perforated transparent acrylic plastic box (7 × 10 × 40 cm) on one side. Social avoidance behavior was evaluated based on a 2-stage SIT (a video recording tool was used for recording). In the first stage (“target-absent”), all C57 mice were placed in the arena under 5 lux and allowed to explore freely for 150 s without a CD1 aggressor mouse in the interaction zone (IZ). At the end of the first phase, the experimental mice were removed for 30 s, and the arena was cleaned. Next, a CD1 mouse was placed in a transparent plastic box, and the test mouse was reintroduced into the arena. Then, the second stage of the test was performed, and the same metrics were measured for 150 s. The time spent in the IZ in the presence and absence of the target was recorded.

### Sucrose Preference Test

In the present study, the SPT was conducted, as discussed earlier (Jiang et al., 2020a). On the first 2 days (at 9:00 a.m.), each mouse was concurrently presented with two bottles containing 100 ml of either 1% sucrose solution or tap water. The animals had access to food/liquids ad libitum, and the location of the water bottles was altered every 12 h to minimize potential location bias. On the third day (at 9:00 a.m.), we removed the food and the bottles, followed by 8 h of inaccessibility to water/food. At approximately 17:00 p.m., each mouse was allowed to drink the pre-weighed sugar water and pure water for 16 h in a quiet and peaceful environment. On the next day (at 9:00 a.m.), all the bottles were removed, weighed, and recorded. The sucrose preference was measured by the following equation: sucrose preference index (%) = (sucrose solution consumed/total solution consumed) × 100%.

### Forced Swim Test

The FST was carried out according to the previously reported protocol. All mice were restrained to swim for 6 min at a 15 cm depth in an acrylic cylinder (d × h: 14 × 20 cm) at room temperature. Video tracking software (Tail Suspension Real-Time Analysis System 2.0) was used for the recording of immobility time during the last 4 min post-habituation (2 min) in a 6-min test.

### Western Blotting Analysis

The analysis was carried out according to the previous method with slight modifications (Kim et al., 2018; Xie et al., 2019). First, the mice were euthanized and then rapid dissection of the bilateral whole hippocampus was carried out on the ice, followed by homogenizing in lysis buffer for 0.5 h. After RIPA lysis buffer (comprising phosphatase/protease inhibitors) homogenization, the centrifugation (12,000 g) of the hippocampal homogenate was carried out at 4°C for 15 min and then the BCA protein assay was used for quantification of proteins. Subsequently, SDS-PAGE (10%) was employed for the separation of protein samples (30 μg), followed by transferring onto a PVDF membrane (Millipore, United States). For the blockage purpose of the membrane nonspecific sites, the membranes were incubated with skimmed milk (5%, dry) for 60 min in TBS-T. Then, for 24 h, the membranes were incubated with primary antibodies (at 4°C) *i.e.,* ERK 1/2 (1:2000; 9107#), phospho-ERK1/2 (*p*-ERK1/2; 1:2000; 4370#), CREB (1:500; 9197#), phospho-CREB-Ser133 (*p*-CREB; 1:500; 9198#), AKT (1:2000; 4691#), phospho-AKT (p- AKT; 1:2000; 4060#), β-actin (1:1000; 4967#) (all from Cell Signaling, United States), and BDNF (1:5000; Ab108319). After washing, the membranes were incubated with secondary antibodies (HRP-conjugated) at ∼ 25°C for 60 min. The bands were visualized *via* enhanced chemiluminescence and were captured *via* ChemiDoc XRS (Bio-Rad, United States).

### Immunofluorescence Studies

Immunofluorescence studies were performed following the reported method with slight modifications [30, 31]. Following the behavioral tests, the animals were anesthetized by injecting pentobarbital sodium, followed by the transcardial perfusion *via* paraformaldehyde (4%) in phosphate buffer (0.01 M) for 24 h after the last session. The removal of the brain was carried out carefully and then the brain was post-fixed with the formaldehyde (4%) fixative and embedded in paraffin, followed by dissecting into thick sections (5 µm).

For double fluorescence staining, the sections were successively exposed to Triton X-100 (0.3%) in PBS (0.01 M) for 0.5 h and BSA (3%) in PBS (0.01 M) for 0.5 h. Next, the sections were incubated with a mouse anti-NeuN antibody (1:100; Ab77450#) and a rabbit anti-DCX antibody (1:100; Ab177487#) at 4°C for 24 h (Abcam, United Kingdom). The sections were subsequently exposed to fluorescein isothiocyanate–labeled goat anti-rabbit IgG (1:300; GB22303) and rhodamine-labeled goat anti-mouse IgG (1:300; GB22301) for 50 min (Servicebio). PBS (0.01 M) was used for washing the sections, followed by their fixation on slides. After fixation, the slides were subjected to dehydration and were coverslipped. A fluorescence microscope (Leica, Germany Q9) was used for visualizing images with ×400 magnification. ImageJ software (Media Cybernetics, United States) was used for evaluating the fluorescence intensity of each group.

### Data and Statistical Analyses

The analysis of the obtained results was performed *via* SPSS Statistics version 21.0 (Chicago, United States). The variations between mean values were determined *via* one-way or two-way ANOVA, as appropriate. For all one-way ANOVA analyses, post hoc tests were performed using the least significant difference test. For all two-way ANOVA analyses, Bonferroni post hoc tests were used to assess isolated comparisons. A *p*-value less than 0.05 was considered statistically considerable, and the obtained values were indicated as mean ± SEM.

## Results

### Rb1 Treatment Prevents the Development of Depressive-Like Behaviors in CSDS-Exposed Mice

**FIGURE 2:**
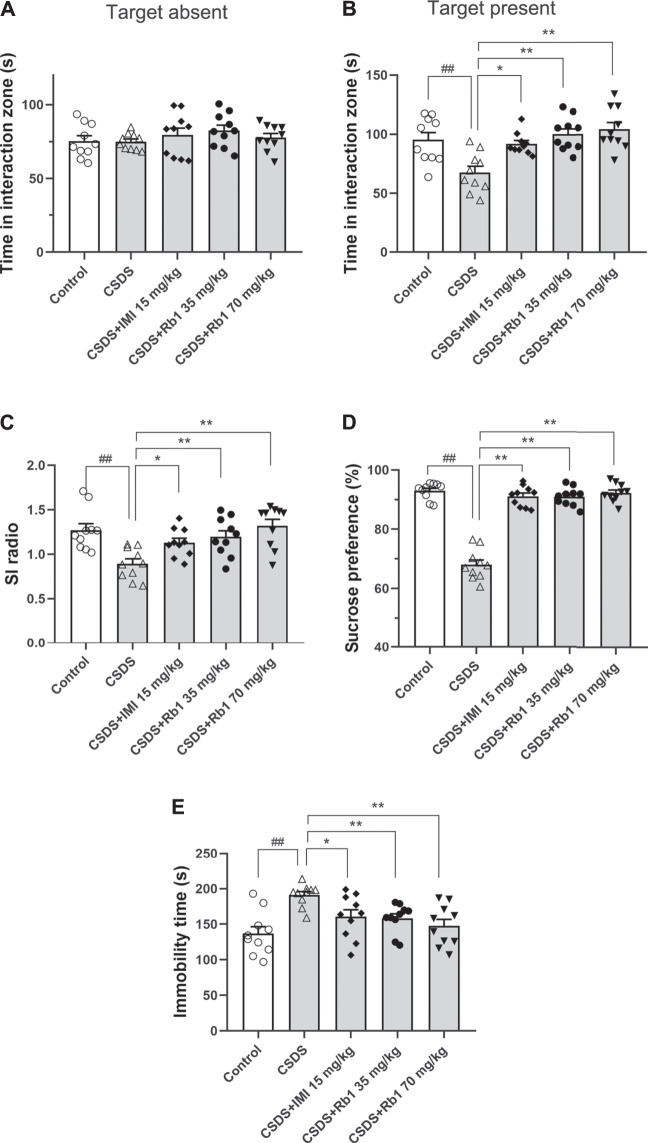
Rb1 exerted antidepressant-like effects in a CSDS-induced depression model **(A–C)** The time spent in the social IZ and the SI ratio in the SIT. **(D)** The intake of sucrose (in percentage) in the SPT. **(E)** Immobility time in the FST. N = 10 in each group; the data are presented as the mean ± S.E.M. ^##^ *p* < 0.01, vs. the control group; ^*^ *p* < 0.05, **p < 0.01, vs. the group exposed to CSDS; one-way ANOVA.

After 28 consecutive days of modeling and drug administration, different depressive-like behaviors were evaluated. These behaviors included anhedonia, as assessed by the sucrose preference test (SPT); social avoidance, as measured by the time spent in the IZ; and behavioral despair, as evidenced by the immobility time in the forced swim test (FST). In the vehicle-treated CSDS-exposed group, significant declines in the time spent in the IZ and social interaction (SI) ratio in the SIT (F_4,45_ = 5.903, *p* < 0.01; F_4,45_ = 6.500, *p* < 0.01) and sucrose consumption (F_4,45_ = 31.747, *p* < 0.01) and an increase in the immobility time in the FST (F_4,45_ = 6.109, *p* < 0.01) were observed, as shown in Figures 2A–E, respectively. However, the time spent in the IZ (*p* < 0.01 and <0.05) and sucrose intake in the SPT (*p* < 0.01 and 0.05) were considerably increased, and the immobility time in the FST (*p* < 0.01 and <0.05) was decreased in the groups treated with Rb1 (35 or 70 mg/kg) compared with the CSDS-exposed group; similar changes were seen in the 15 mg/kg IMI-treated group (*p* < 0.05). These results reveal that Rb1 exerts a potent antidepressant-like effect based on these behavioral tests.

### Rb1 Treatment Counteracts the CSDS-Induced Deficits in Hippocampal Neurogenesis

**FIGURE 3:**
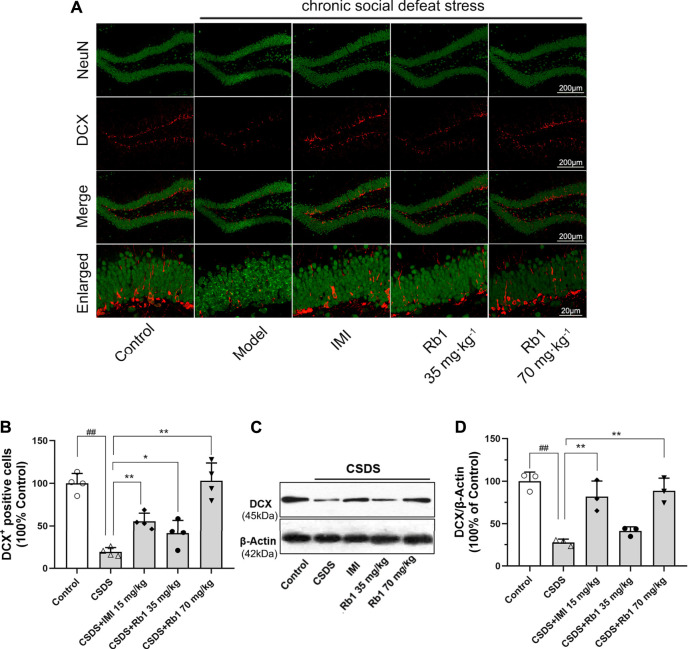
Rb1 treatment improved the hippocampal neurogenesis in the CSDS depression model. **(A)** Representative immunofluorescence images of the dentate gyrus (DG) and costaining of the neuronal nuclear protein (NeuN) and DCX in green. The scale bar is 200 and 20 μm for the representative and the enlarged images, accordingly. **(B)** The number of DCX + cells in the DG. **(C–D)** Represents immunoblotting of DCX coincided with immunohistochemical changes. N = 3–4 per group, the obtained data have been depicted as the mean ± S.E.M. ^##^ *p* < 0.01, vs. the control group; ^*^ *p* < 0.05, **p < 0.01, vs. the model group.

It has been revealed that chronic stress lowers HN, which contributes to the onset of depression. The immunoreactivity of hippocampal doublecortin (DCX) was determined to evaluate the influence of Rb1 on HN. DCX is a microtubule-associated protein that is transiently expressed in newborn neurons and is used as a marker of neurogenesis (Jiang et al., 2014). Immunofluorescence revealed that the expression level of DCX was decreased in the dentate gyrus (DG) (red fluorescence) after 28 days of exposure to CSDS and that this change was reversed by Rb1, as presented in Figure 3A. Upon exposure to CSDS, the mice showed a marked reduction in the number of DCX^+^ cells in the DG (F_4,15_ = 44.883, *p* < 0.01), as shown in Figure 3B. Moreover, treatment with Rb1 (35 or 70 mg/kg) or IMI (15 mg/kg) for 33 days significantly increased the number of DCX^+^ cells in the DG (*p* < 0.01 and 0.01, respectively). Similarly, Western blotting revealed a significant decline in DCX protein expression in the hippocampus of the CSDS-exposed mice (F_4,10_ = 21.265, *p* < 0.01), as shown in Figure 3C, whereas Rb1 (35 and 70 mg/kg) treatment counteracted the decrease in BDNF expression induced by CSDS (*p* < 0.01, each). These findings suggest that Rb1 has protective effects on adult HN.

### Rb1 Treatment Reverses the CSDS-Induced Inhibition of the BDNF Signaling Cascade

**FIGURE 4:**
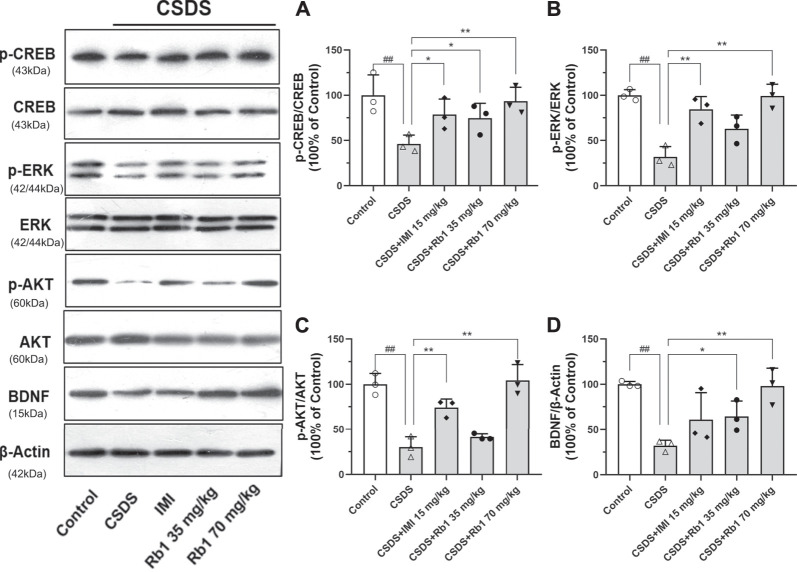
Influence of Rb1 treatment on the expression of the BDNF signal pathway in the hippocampus of mice exposed to CSDS. **(A)** pCREB/CREB, **(B)** pERK/ERK, **(C)** pAKT/AKT, and **(D)** BDNF protein levels. N = 3 per group, the results are indicated as the mean ± S.E.M. ^##^ *p* < 0.01, vs. the control group; ^*^ *p* < 0.05, **p < 0.01, vs. the group exposed to CSDS, by one-way ANOVA analysis.

BDNF is a neurotrophic factor (NF) that substantially contributes to neurogenesis in adults and is thus associated with the pathogenesis of depression (Bjorkholm and Monteggia, 2016). In the current study, it was hypothesized that Rb1 may increase the expression of hippocampal BDNF. The Western blotting results revealed that hippocampal BDNF protein expression was considerably increased by exposure to Rb1, particularly at a dose of 70 mg/kg (Figure 4D; F_4,10_ = 7.543, *p* < 0.01). As indicated in Figures 4A–C, Rb1 (70 mg/kg) elevated the levels of the phosphorylated and activated forms of ERK (F_4,10_ = 16.244, *p* < 0.01), CREB (F_4,10_ = 4.660, *p* < 0.01), and AKT (F_4,10_ = 24.889, *p* < 0.01), which have been associated with activation of BDNF signaling.

### BDNF–TrkB Signaling May Significantly Contribute to the Antidepressant Effect of Rb1

**FIGURE 5:**
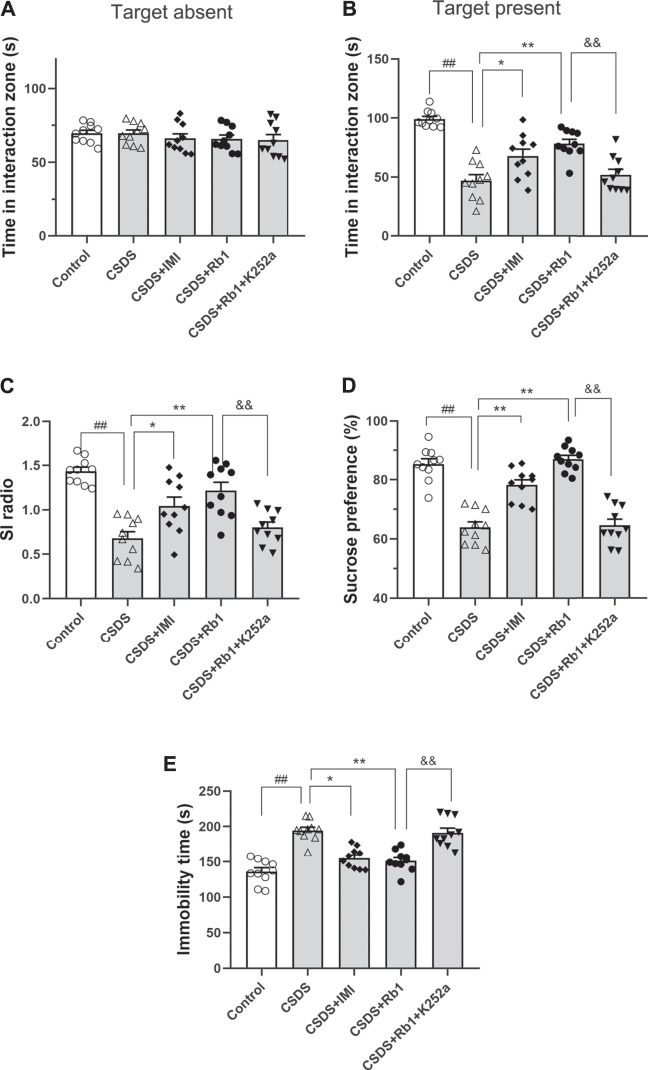
Blockage of BDNF–TrkB signaling by K252a abolished the antidepressant activity of SY. **(A–C)** The time in the social interaction zone and the social interaction (SI) ratio in the SIT. **(D)** The intake of sucrose (in percentage) in the SPT. **(E)** Immobility time in the FST. The obtained results are indicated as means ± SEMs (n = 10). ^#^ *p* < 0.05, ^##^ *p* < 0.01, vs. the control group; ^*^ *p* < 0.05, **p < 0.01, vs. the model group.

**FIGURE 6:**
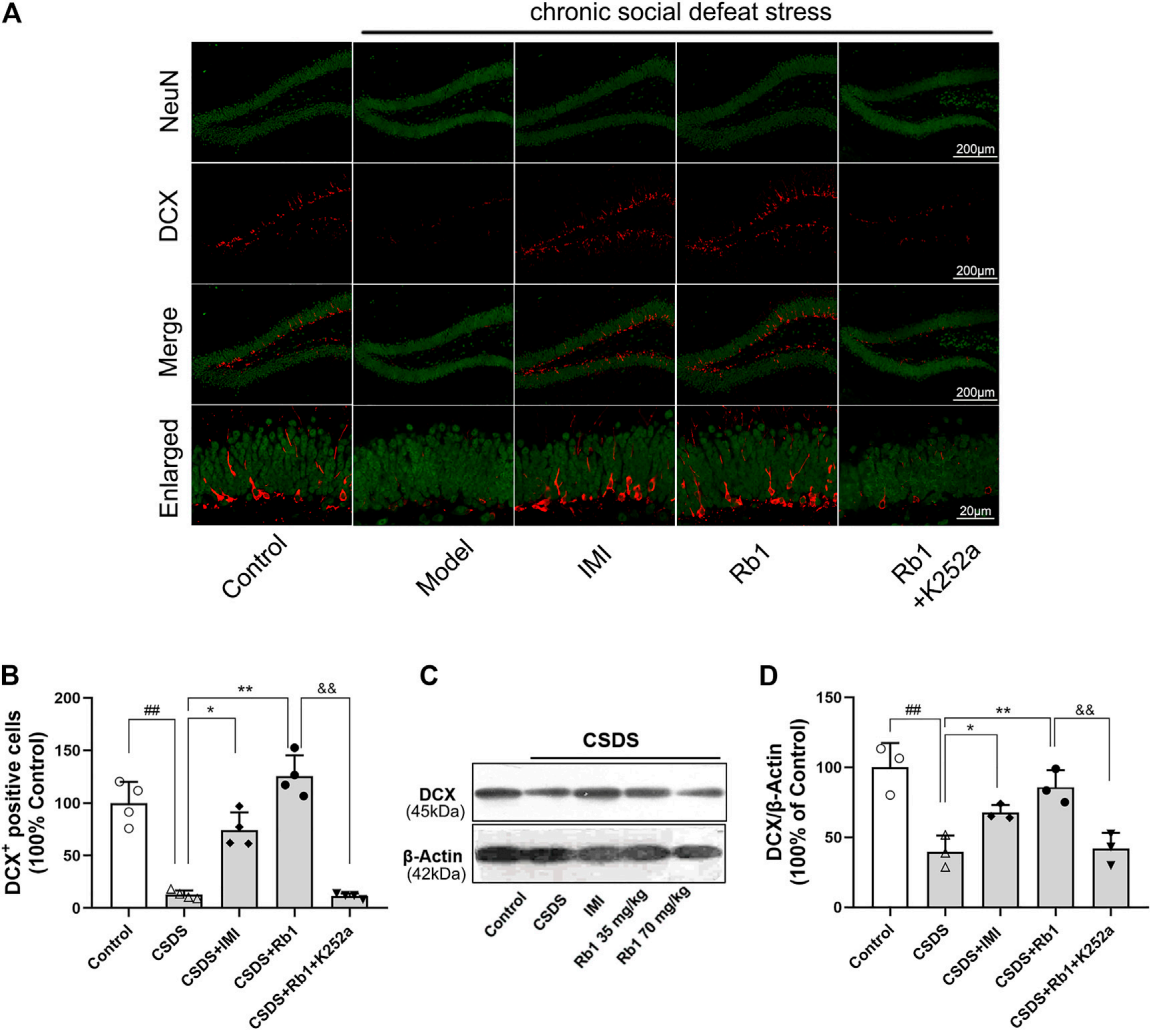
Impact of pretreatment with K252a on the number of DCX-positive cells (Rb1-induced) in CSDS. **(A)** Representative immunofluorescence images of the dentate gyrus (DG) and costaining of the neuronal nuclear protein (NeuN) and DCX in green color. The scale bar is 200 and 20 μm for the representative and the enlarged images, accordingly. **(B)** The number of DCX + cells in the DG. **(C–D)** Represents immunoblotting of DCX coincided with immunohistochemical changes. N = 3–4 per group; the obtained data have been depicted as mean ± S.E.M. ^#^ *p* < 0.05, ^##^ *p* < 0.01, vs. the control group; ^*^ *p* < 0.05, **p < 0.01, vs. the model group.

**FIGURE 7:**
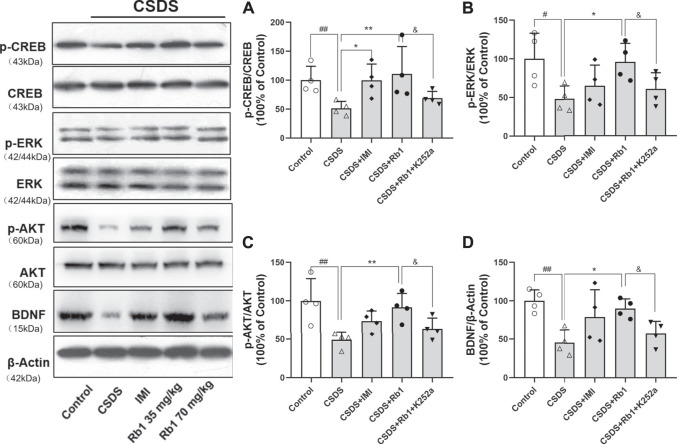
Impact of pretreatment with K252a on Rb1-activated BDNF. The influence of SY on the pCREB/CREB **(A)**, pERK/ERK **(B)**, pAKT/AKT **(C)**, and BDNF **(D)** protein levels in the hippocampus of the rats exposed to CUMS. The results have been indicated as means ± SEMs (n = 4). ^#^ *p* < 0.05, ^##^ *p* < 0.01, vs. the control group; ^*^ *p* < 0.05, **p < 0.01, vs. the model group.

In the current study, K252a, which potently inhibits the BDNF receptor, that is, TrkB, was used. In brief, K252a (25 μg/kg) was injected into mice exposed to CSDS, Rb1 (70 mg/kg) was administered to these mice, and then behavioral tests were conducted. As presented in Figure 5, K252a abolished the antidepressant effects of Rb1 (70 mg/kg) in CSDS-exposed mice in the SIT, SPT, and FST. Similarly, Figures 6 and 7 show that K252a not only blocked the effects of Rb1 on neurogenesis, as indicated by the fact that Rb1 treatment did not restore the number of DCX^+^ cells in the DGs of the mice (exposed to CSDS), but also prevented the effects of Rb1 on the expression of BDNF, *p*-CREB, *p*-ERK1/2, and *p*-AKT in the hippocampi of the CSDS-exposed mice. Together, these results suggest that the BDNF–TrkB signaling cascade is required for the antidepressant effects of Rb1.

## Discussion

In the current study, it was revealed that Rb1 exerts an antidepressant-like effect in a CSDS-induced depression model. Chronic treatment with Rb1 might reverse the reduction in HN and the hippocampal BDNF signaling pathway induced *via* CSDS. By using inhibitors of BDNF–TrkB signaling, we further confirmed that the BDNF signaling cascade is required for the antidepressant-like potential of Rb1. Limitations still exist in the current study. In particular, the results of Western blotting must be considered very preliminary although promising due to the very low number of samples in each experimental group. Therefore, more studies involving more animals are required to confirm the results of Western blots and to understand the mechanisms of the antidepressant effects of Rb1. Moreover, Rb1 has been demonstrated its protection in the central nervous system and is apparently highly distributed to the brain. Previous studies reported that Rb1 could cross the blood–brain barrier (BBB) wnter the brain and the specific distribution of Rb1 in the rat brain (Li et al., 2007). Recent studies indicate that the transport of Rb1 at the BBB is at least partly mediated by the GLUT1 transporter *in vitro* and *in vivo* (Wang GL. et al., 2018). More insights into the pharmacokinetic properties of Rb1, especially for the blood–brain barrier permeability of Rb1, could be useful for its development as a suitable treatment and should be considered in more studies.

Animal models of CSDS-induced depression are useful and effective models that are widely used for studying psychosocial stress–induced depression (Yin et al., 2015). Being consistent with earlier results, the findings of this study showed that CSDS persistently induced a number of depression-like phenotypes characterized by despair, anhedonia, and social-avoidance behaviors, as demonstrated by deficits in the SIT, reduced consumption of sucrose solution in the SPT, and increased immobility time in the FST (Jiang et al., 2019b). These CSDS-induced changes in behavior were ameliorated by long-term treatment with Rb1 (35 or 70 mg/kg), which had a similar effect as the classical antidepressant IMI, suggesting that Rb1 may be a novel candidate for the treatment of depression.

In humans and several other species, neurogenesis in the hippocampal region starts postnatally and continues into adulthood. The development of hippocampal neurons is highly vulnerable to the adverse effects of stress and is involved in the pathophysiology and treatment of mood disorders (Jiang et al., 2019b). A reduction in neurogenesis may significantly contribute to depressive episodes, and an improvement in HN has been correlated with the use of antidepressants (Sánchez-Vidaña et al., 2019). In the current study, 4 weeks of CSDS exposure suppressed neurogenesis in the mouse DG, as shown by a significant decrease in the number of DCX^+^ cells, which is consistent with our earlier studies (Jiang et al., 2020b). However, treatment with Rb1 (35 or 70 mg/kg) elevated the number of DCX^+^ cells in the DG and reversed the CSDS-induced suppression of neurogenesis, revealing that Rb1 may be a potential pro-neurogenic drug.

The obtained results also indicated that Rb1 elevated BDNF protein expression in the hippocampi of CSDS mice. BDNF is a key NF that regulates cell survival, substantially contributes to adult neurogenesis, and is important for the pathogenesis and treatment of depression (Warner-Schmidt and Duman, 2006; Bjorkholm and Monteggia, 2016). It was found that BDNF levels are considerably decreased in the hippocampi and cortices of rodents upon exposure to chronic stress; conversely, BDNF expression in these regions is increased by chronic treatment with antidepressants and is required for the effects of drugs on behaviors (Ghosal et al., 2018). Several studies have revealed that Rb1 regulates the expression of BDNF and activates neurogenesis in rats with experimental cerebral ischemia (Gao et al., 2010). Recent studies have shown that Rb1 pretreatment reverses the changes in BDNF/TrkB mRNA and protein expression in the hippocampus in rats exposed to acute immobilization stress (Kang et al., 2019). We thus speculate that Rb1 may promote downstream signaling cascades of BDNF, improve neurogenesis, and exert antidepressant effects. CSDS considerably decreased BDNF expression in the hippocampus, which was consistent with earlier results (Jiang et al., 2017). Conversely, Rb1 prevented the CSDS-stimulated reduction in hippocampal BDNF expression, which corresponded to elevation of neurogenesis. It has been demonstrated that BDNF phosphorylates and activates the protein CREB in the nucleus *via* the TrkB receptor, subsequently promoting the downstream MAPK/ERK and PI3K/AKT signaling cascades, which regulate the growth and survival of neuronal cells in the hippocampus, mediate depression (induced *via* stress), and exert antidepressant effects (Luo et al., 2015; Wu et al., 2018). In the current study, it was revealed that Rb1 treatment enhanced the phosphorylation of AKT and ERK1/2, two downstream regulators of BDNF, in the hippocampus and alleviated depression symptoms in CSDS mice. Moreover, K252a blocked the antidepressant effects of Rb1 in the behavioral tests and enhanced neurogenesis, confirming that the activation of BDNF signaling is required for the antidepressant-like activity of Rb1.

## Conclusion

In brief, the current study revealed that Rb1 prevents depression-like symptoms in socially defeated mice, which seems to be facilitated *via* activation of the hippocampal BDNF–TrkB signaling pathway. The study explored the pharmacological properties of Rb1 and revealed that Rb1 could be an important candidate molecule in the prevention and treatment of disorders associated with stress, including depression, exerting considerable effects and relatively few adverse effects.

## Data Availability

The raw data supporting the conclusion of this article will be made available by the authors, without undue reservation.
